# Customized liver organoids as an advanced *in vitro* modeling and drug discovery platform for non-alcoholic fatty liver diseases

**DOI:** 10.7150/ijbs.85145

**Published:** 2023-07-09

**Authors:** Dong Wook Han, KangHe Xu, Zhe-Long Jin, Yong-Nan Xu, Ying-Hua Li, Lin Wang, Qilong Cao, Kee-Pyo Kim, DongHee Ryu, Kwonho Hong, Nam-Hyung Kim

**Affiliations:** 1 Guangdong Provincial Key Laboratory of Large Animal Models for Biomedicine, School of Biotechnology and Health Sciences, Wuyi University, Jiangmen, China.; 2 International Healthcare Innovation Institute (Jiangmen), Jianghai, Jiangmen, Guangdong Province, China.; 3 Research and Development, Qingdao Haier Biotech Co. Ltd, Qingdao, China.; 4 Guangdong ORGANOID Biotechnology Co. Ltd, Jiangmen, China.; 5 Department of Surgery, College of Medicine, Chungbuk National University, Cheongju, Republic of Korea.; 6 Department of Life Sciences, College of Medicine, The Catholic University of Korea, Seoul, Republic of Korea.; 7 Department of Stem Cell and Regenerative Biotechnology, The institute of advanced regenerative science, Konkuk University, Seoul, Republic of Korea.

**Keywords:** non-alcoholic fatty liver disease (NAFLD), non-alcoholic steatohepatitis (NASH), liver organoids, disease modeling, drug discovery

## Abstract

Non-alcoholic fatty liver disease (NAFLD) and its progressive form non-alcoholic steatohepatitis (NASH) have presented a major and common health concern worldwide due to their increasing prevalence and progressive development of severe pathological conditions such as cirrhosis and liver cancer. Although a large number of drug candidates for the treatment of NASH have entered clinical trial testing, all have not been released to market due to their limited efficacy, and there remains no approved treatment for NASH available to this day. Recently, organoid technology that produces 3D multicellular aggregates with a liver tissue-like cytoarchitecture and improved functionality has been suggested as a novel platform for modeling the human-specific complex pathophysiology of NAFLD and NASH. In this review, we describe the cellular crosstalk between each cellular compartment in the liver during the pathogenesis of NAFLD and NASH. We also summarize the current state of liver organoid technology, describing the cellular diversity that could be recapitulated in liver organoids and proposing a future direction for liver organoid technology as an *in vitro* platform for disease modeling and drug discovery for NAFLD and NASH.

## Introduction

The liver is the largest solid organ in the human body and plays critical roles in a wide range of physiological functions, including metabolism, detoxification, and protein production 1, 2. The liver is composed of several cell types, including 1 hepatocytes, the parenchymal cells of the liver 2; cholangiocytes, the epithelial cells of the bile ducts 3; Kupffer cells, the resident macrophages 4; hepatic stellate cells (HSCs) that store vitamin A and produce extracellular matrix (ECM); and 5 highly specialized endothelial cells known as liver sinusoidal endothelial cells (LSECs) 1, 3. The cell-to-cell communication among these cell types is essential for maintaining functional homeostasis of liver 4.

The liver is susceptible to many types of damage, and long-term liver damage can lead to chronic liver diseases 5. Among chronic liver diseases, non-alcoholic fatty liver disease (NAFLD), one of the most common liver diseases 6, 7, is characterized by the excessive accumulation of triglycerides in liver cells exceeding 5% of the liver's weight 8. While NAFLD can be a simple steatosis, it can potentially develop into non-alcoholic steatohepatitis (NASH), the most severe form of NAFLD 9. NASH can further develop into cirrhosis, liver cancer, and liver failure, and becomes a leading indication for liver transplantation 10. The global prevalence of NAFLD has been rapidly increasing and its current global prevalence is estimated to be 25% 11. Thus, NAFLD and NASH have become a major global health concern.

For the past couple of decades, many pharmaceutical companies have undertaken tremendous efforts for developing new drugs for NAFLD and NASH 12. However, these efforts have failed to achieve novel and effective treatment for NAFLD and NASH 13. Although animal models have enhanced our fundamental understanding of the pathogenesis of NAFLD and NASH, a growing body of evidence has suggested that animal models are not sufficient for translating scientific findings from animals into humans due to the fundamental genetic and physiological differences between animals and humans, necessitating the development of a human-specific model system 14, 15. Primary human hepatocytes (PHHs), a gold standard for hepatic research, have also faced large hurdles, such as their limited accessibility and rapid loss of functionality upon *in vitro* culture, impeding their industrial and clinical application 16, 17. As an alternative to PHHs, 2D hepatocyte-like cells generated from human pluripotent stem cells (hPSCs) and directly converted induced hepatocytes (iHeps) with defined factors have been also suggested 18-23. However, the relatively low functionality and proliferative capacity of these 2D hepatocyte-like cells has also hampered the translation of these cell types into the human setting 24. Furthermore, previous 2D cell types including PHH and 2D hepatocyte-like cells lack pro-fibrotic and pro-inflammatory cell types, which are critical for initiation and progression of NAFLD and NASH 15. Therefore, developing a human-specific model with liver tissue-like cell type composition and cytoarchitecture is direly needed for closely mirroring the pathogenesis of NAFLD and NASH.

To overcome the aforementioned issues of previous model systems, recent organoid technology was utilized for generating 3D liver tissue-like organoids (liver organoids) with improved structural and functional features. The stably expandable liver organoids displaying structural and functional similarities with liver tissue could be robustly generated from both liver biopsy and hPSCs 14, 25, 26. Moreover, recent advances have also described the presence of not only parenchymal cell types including both hepatocytes and cholangiocytes but also non-parenchymal cell types including HSCs and Kupffer cells 27, 28 with a functional bile canaliculi network in liver organoids 29. However, liver organoids generated using different protocols exhibit quite diverse structural and functional features with distinct cellular makeups. Considering the roles of each cell type (hepatocytes, cholangiocytes, HSCs, Kupffer cells, and LSECs) in the initiation and progression of NAFLD and NASH, the development of liver organoid models with *in vivo-*like cell type diversity is a prerequisite for future research examining *in vitro* modeling and drug discovery for NAFLD and NASH.

In the current study, we describe the role of each liver cell type and the cellular crosstalk among these diverse cell types in the pathogenesis of NAFLD and NASH. We next summarize current technical advances of liver organoid technology and compare the structural and functional features of liver organoids generated using distinct protocols. We also discuss the potential usefulness as well as the limitation of liver organoid technology as an *in vitro* modeling and drug screening platform for NAFLD and NASH. Finally, we propose a novel concept for utilizing customized liver organoids that properly recapitulate key inter-cellular, inter-tissue, and inter-organ communications for the pathogenesis of NAFLD and NASH. The distinct types of customized liver organoids such as monocellular liver organoids, multi-tissue liver organoids, and multi-organ liver organoids might represent a novel and suitable *in vitro* model system for unveiling the underlying mechanism of NAFLD and NASH and for discovering novel therapeutics.

## 1. About NAFLD and NASH

NAFLD is the most common liver disease worldwide, affecting approximately 25% of the world's population 30. The incidence has increased by 7.5% per year over 10 years, especially in young adults (<45 years) 31. Indeed, among adults aged 18 to 39 years, the prevalence of NAFLD has increased by about seven-fold 32. A total of 6% to 30% of individuals diagnosed with NAFLD by ultrasound could see their disease develop into NASH with biopsy confirmation 30, eventually leading to severe liver fibrosis, cirrhosis, and hepatocellular carcinoma (HCC) 10. Due to the high prevalence of NAFLD, the incidence of HCC associated with NASH has also been increasing and, subsequently, HCC has become the fourth leading cause of cancer death worldwide 33. Indeed, patients with NAFLD or NASH show a significantly higher incidence of HCC compared to unaffected individuals 30, 34. Thus, the number of registered patients requiring liver transplantation due to NASH has been rapidly increasing 35.

NAFLD is a spectrum of liver diseases characterized by hepatic steatosis without liver damage or inflammation, and it is usually associated with obesity 36. In contrast, NASH is a more severe form of NAFLD, with the main pathological features including steatosis, inflammation, and fibrosis 37. The pathogenesis of NAFLD without the influence of alcohol is complex and associated with multiple factors including genetic and epigenetic factors, metabolism, and the gut-liver axis, among others 38. Excessive lipid accumulation leads to hepatocyte lipotoxicity, which is an important link in the development of NAFLD 37. It was demonstrated that insulin resistance promotes the release of free fatty acids (FFA) from adipose tissue into the blood and that insulin induces the production of new FFA in the liver through new lipogenesis, where these FFA recombine to form triglycerides, the main component of fat accumulated in the liver 39. Insulin resistance also promotes adipose tissue dysfunction, which alters the production and secretion of adipokines and inflammatory cytokines 40. The damaged hepatocytes also secrete inflammatory cytokines and chemokines and their cellular contents, which activate Kupffer cells, resulting in a pro-inflammatory response and cellular immune infiltration, leading to the development of NASH 41. Once NAFLD has progressed to NASH, it further promotes insulin resistance in adipose tissue and the liver, which leads to a harmful cycle of insulin resistance, liver fat accumulation, and inflammation 39. The accumulation of fat in the liver in the form of triglycerides also leads to mitochondrial dysfunction, activation of oxidative stress, reactive oxygen species (ROS) production, and endoplasmic reticulum (ER) stress-related mechanisms, ultimately leading to hepatocyte death 41.

**Figure 1:**
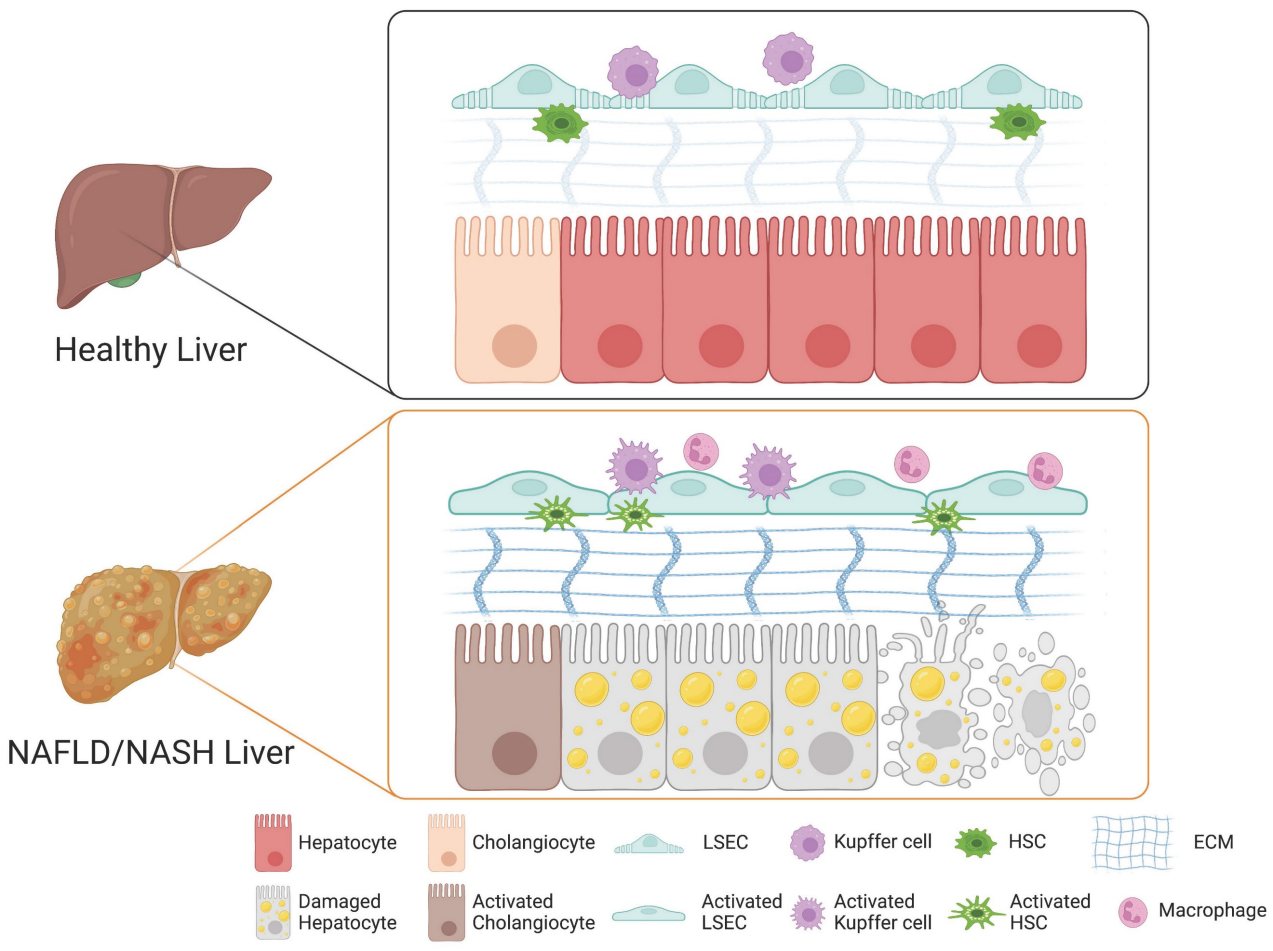
Role of each cell type in the liver during the pathogenesis of NAFLD and NASH. In a healthy liver, hepatocytes, cholangiocytes, Kupffer cells, HSCs, and LSECs play distinct roles in maintaining both structural and functional homeostasis. Upon injury, the cellular crosstalk among distinct hepatic cellular compartments leads to drastic structural and phenotypic alterations and to activation of cholangiocytes, LSECs, Kupffer cells, and HSCs into their relevant pathological states, triggering the pathogenesis of NAFLD and NASH.

A growing body of evidence suggests that the cellular crosstalk among distinct cellular compartments in the liver is crucial for the initiation and progression of NAFLD and NASH. Although the underlying mechanism of NASH pathogenesis remains largely elusive, each non-parenchymal liver cell compartment, including Kupffer cells, HSCs, and LSECs, is known to trigger hepatocyte injury, inflammation, fibrosis, and vascular dysfunction. Here, we describe the role of each non-parenchymal cell type in the pathogenesis of NAFLD and NASH (Figure 1).

### 1) Role of Kupffer cells in NASH

Liver macrophages consist mainly of two populations, Kupffer cells and monocyte-derived macrophages 42. Kupffer cells are the resident macrophages in the lumen of hepatic sinusoids and account for 80-90% of colonized macrophages in the human body 43. Kupffer cells play a crucial role in regulating and maintaining immunity in the liver 43. Upon different stimuli, macrophages including Kupffer cells and monocyte-derived macrophages undergo phenotypic differentiation into either classically activated M1 macrophages or alternatively activated M2 macrophages 44. M1 macrophages with a high antigen presentation capacity produce diverse pro-inflammatory cytokines including TNFα, IL-1B, CCL2, and CCL5 and promote macrophage-mediated tissue damage. In contrast, M2 macrophages are involved in anti-inflammatory responses and tissue repair via balancing the activity of M1 macrophages 45. In NAFLD, the balance between pro-inflammatory M1 Kupffer cells and anti-inflammatory M2 Kupffer cells is critical for modulating the initiation and progression of liver injury 46. Indeed, a previous study demonstrated that the selective cell death of M1 Kupffer cells leads to M2 Kupffer cell-mediated protection of alcoholic liver injury 47. Therefore, balancing pro-inflammatory M1 Kupffer cells and anti-inflammatory M2 Kupffer cells would be an alternative strategy for blocking further progression of NAFLD 47.

In the case of NASH, damaged hepatocytes lead to activation of Kupffer cells and infiltration of circulating monocyte-derived macrophages (Figure 1) 48. The activated macrophages produce pro-inflammatory cytokines that induce the activation and transdifferentiation of HSCs and also influence the physiological functions of other cellular compartments such as LSECs and other immune cells 49, 50. The crosstalk between Kupffer cells and hepatocytes is bidirectional 41. The damaged hepatocytes release their cellular contents including damage-associated molecular patterns (DAMPs), contributing the activation of Kupffer cells 51. The activated Kupffer cells produce diverse cytokines, contributing to hepatic lipid deposition and hepatocyte death 52. On the other hand, Kupffer cells also exert their roles in the elimination of apoptotic hepatocytes via efferocytosis, by which DAMP-mediated inflammation can be ameliorated by clearing DMAP-producing damaged hepatocytes 53. An increasing body of evidence suggests the crucial roles of Kupffer cells in the progression and regression of NASH.

### 2) Role of HSCs in NASH

HSCs represent 5-8% of all liver cells 54. HSCs reside in the space of Disse, a thin perisinusoidal area between sinusoidal endothelial cells and hepatocytes 55. Under normal physiological conditions, HSCs are in a quiescent state and store vitamin A in lipid droplets 56. Upon liver injury, quiescent HSCs undergo phenotypic switch toward activated HSCs, which are proliferative, migrative, contractile, and fibrogenic, with progressive loss of their vitamin A-storing activity 57. Although HSCs play a central role in the deposition of extracellular matrix (ECM) 58, an intricate macromolecular structural network forming a scaffold for adhesion 59, the deposition and remodeling of ECM in the space of Disse, a key factor for liver fibrosis, is rather orchestrated by cellular crosstalk among multiple cell types 60. Kupffer cells and Kupffer cell-derived cytokines and chemokines play a crucial role in the activation of HSCs and transdifferentiation of these cells into myofibroblasts, subsequently leading to liver fibrosis 61. The release of TGF-β, the most important fibrogenic cytokine, from Kupffer cells activates HSCs via multiple SMAD proteins 62. The Sonic hedgehog pathway is another driving force for HSC activation 63. The accumulation of fat as a form of triglyceride, in the liver causes mitochondrial dysfunction, activation of oxidative stress, production of ROS, and an ER-stress-related mechanism, all contributing to the activation of HSCs 41. Besides these intracellular signaling pathways, other extracellular factors including nutrients, alcohol, and other toxic compounds delivered with portal blood flow to the space of Disse are also known to be involved in HSC activation 64.

In a healthy liver, quiescent HSCs produce collagen IV and VI into the space of Disse, contributing to ECM homeostasis by providing the proper scaffold for architecture and function 60. During liver injury, quiescent HSCs become activated and transdifferentiated into myofibroblasts, with loss of lipid droplets, increased cellular proliferation, and development of mature rough ER to support the production of ECM fibers and matrix remodeling enzymes 65. Activated HSCs start to produce excessive amounts of ECM, mostly collagens I and III, affecting the mechanical characteristics of tissue and the stiffness of the ECM, characteristically encountered during liver fibrosis (Figure 1) 66. The remodeling of ECM impairs the exchange of nutrients and activates the immune system 67. The overproduction of ECM further activates HSCs and contributes to loss of endothelial fenestrations of LSECs, further exacerbating liver fibrosis 68. Thus, the balance between deposition and remodeling of ECM is the key driver for progression of liver fibrosis. Progressive liver fibrosis is known to contribute to the scarring of the liver, which can further develop into cirrhosis, an end-stage liver disease 69.

### 3) Role of LSECs in NASH

In contrast to other blood vessels, the hepatic sinusoids are specialized vascular structures that lack a basement membrane and instead are lined by fenestrated (porous) endothelial cells, so-called LSECs 70. LSECs have both sinusoidal and abluminal sides and can communicate with hepatocytes and HSCs through the abluminal side 71. Under normal physiological conditions, the fenestrae on LSECs mediate the exchange of plasma, nutrients, lipids, and lipoproteins between the sinusoidal lumen and the space of Disse, allowing only particles smaller than the fenestrae to reach the parenchymal cells 72. Moreover, in a healthy liver, LSECs prevent HSC activation and promote reversion to a quiescent state through VEGF-stimulated nitric oxide (NO) production 73. Upon liver injury, however, fenestrae on LSECs undergo drastic changes in their structural and functional properties 74. Defenestration, a reduction in the number and diameter of fenestrae and formation of a continuous basement membrane of LSECs, so-called capillarization, are characteristics typical of chronic liver diseases (Figure 1) 60. While the structural modification of fenestrae can protect the liver from further damage by restricting toxins approaching the parenchymal cells, the defenestration on LSECs influences hepatocytes by creating a microenvironment lacking nutrients and oxygen 75. The lack of nutrients and oxygen from the blood flow impacts the physiological function of hepatocytes and could lead to further progression of liver injury 75. LSECs also indirectly promote liver fibrosis by secreting pro-inflammatory and pro-fibrotic factors 76. Defenestration, the main characteristic of activated LSECs, precedes fibrogenesis in the liver 77. Defenestration and capillarization of LSECs due to liver injury promote the activation of HSCs, contributing to liver fibrosis through loss of VEGF-stimulated NO production 60. A previous study showed that capillarization of LSECs blocks the transfer of chylomicron remnants to hepatocytes, leading to cholesterol and triglyceride synthesis, promoting steatosis 78. Thus, both remodeling of ECM and structural changes of LSECs such as defenestration and capillarization induce hepatocyte apoptosis, promoting the activation of the immune system and chronic inflammation (Figure 1). Subsequently, chronic inflammation could lead to fibrosis, cirrhosis, and even end-stage HCC 79.

### 4) Role of cholangiocytes in NASH

Hepatocytes and cholangiocytes are two main cell types in the liver. While cholangiocytes were mainly known to modulate bile secretion, they have become increasingly recognized for their impact on biliary and liver diseases 80. A previous study has suggested that steatosis can promote biliary senescence and liver fibrosis during cholestasis 81. Cholestasis is a liver disease caused by the reduction or stoppage of bile flow from the liver into the bile duct, leading to hepatic bile acid accumulation and subsequent damage 82. Under normal physiological conditions, cholangiocytes are mitotically quiescent 83. Under pathological conditions, however, they become proliferative, pro-inflammatory, pro-fibrotic, or senescent in response to damage such as cholestasis 84. Senescent cholangiocytes are known to play an important role in liver inflammation and fibrosis during cholestatic liver injury via secretion of cytokines and fibrotic factors such as TGF-β1 and IL-6 80. TGF-β1 is known to activate HSCs and induce their transdifferentiation into myofibroblasts, contributing to liver fibrosis 62. It was previously described that cellular senescence and damage to cholangiocytes in patients with NAFLD and NASH increase with the progression of hepatic steatosis 85. Thus, senescence of cholangiocytes may be an important factor in the progression of NAFLD and NASH.

## 2. Current advances of liver organoid technology

**Figure 2:**
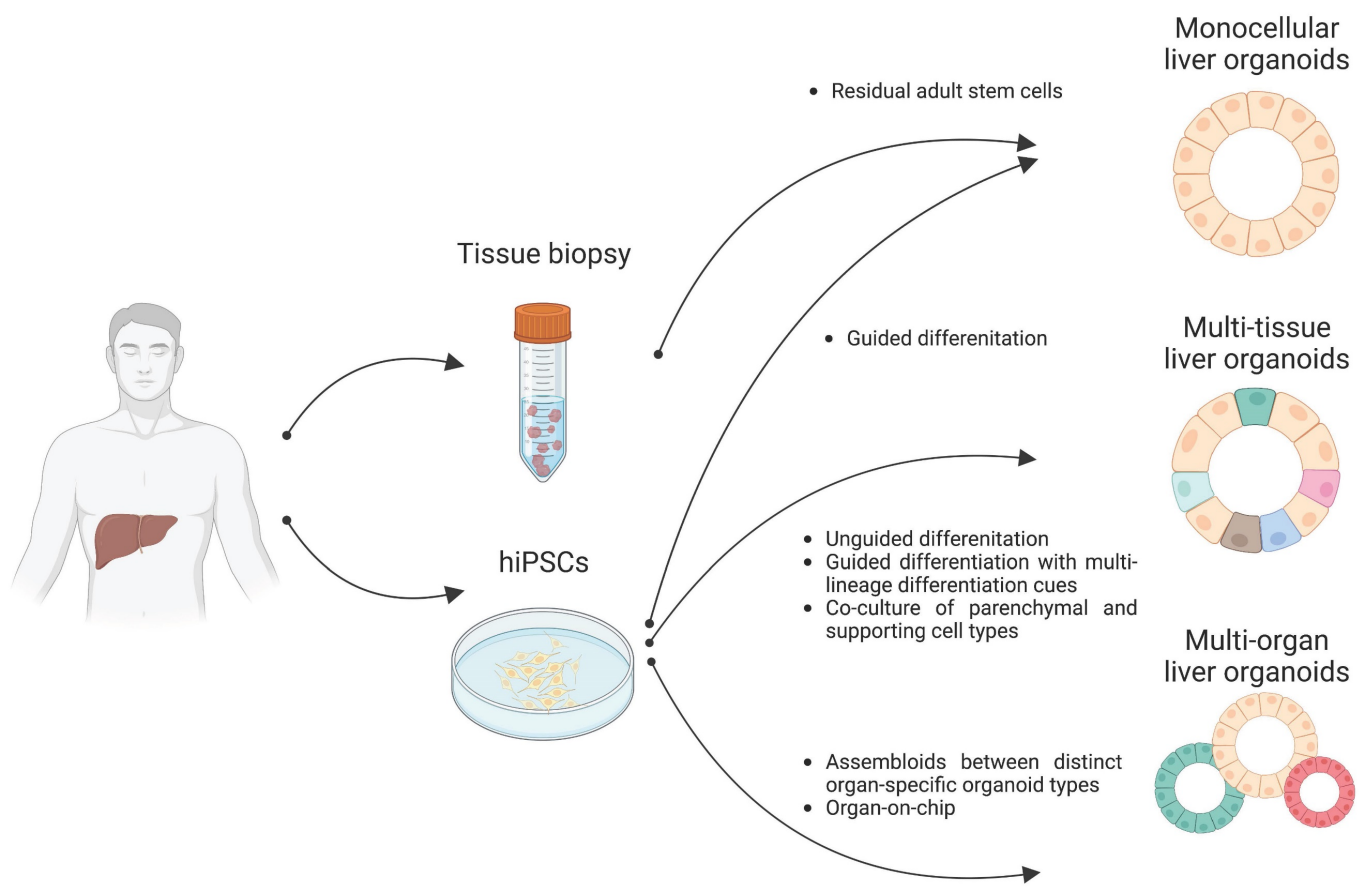
Distinct types of liver organoids. Monocellular liver organoids such as HOs and COs can be generated from either liver tissue or hPSCs. Multi-tissue liver organoids containing both parenchymal and non-parenchymal cell types can be generated by multiple ways such as by unguided differentiation, guided differentiation with multi-lineage differentiation cues, and coculture of parenchymal- and non-parenchymal-supporting cell types. Multi-organ liver organoids with proper inter-organ crosstalk can also be generated by either assembloid or organ-on-chip technology.

Organoids are 3D multicellular aggregates derived from diverse *in vitro* and *in vivo* sources including pluripotent stem cells, multipotent tissue-specific stem cells, and tissue biopsy containing adult stem cells or differentiated cells via cell-to-cell and cell-to-matrix interactions 86, 87. Organoids exhibit structural and functional features comparable to their tissue of origin 24, 88. Early liver organoids typically comprised a monocellular epithelial type such as parenchymal cells (e.g., hepatocytes or cholangiocytes) 14, 25, 26. Recently, multi-tissue liver organoids containing both parenchymal cell types and non-parenchymal supporting cell types such as Kupffer cells and HSCs have been also described 27, 28. Furthermore, recent progress has reported the production of multi-organ organoids interconnecting distinct organ domains in an individual organoid structure, which may be a useful source for understanding liver organogenesis 89 (Figure 2).

As we discussed in the previous section, the pathogenesis and progression of NAFLD and NASH are mediated by a tight cellular crosstalk among distinct liver cell types. Therefore, to establish liver organoid-based human-specific models for NAFLD and NASH, we should clearly understand the current technical situation of liver organoid technology. In this section, we revisit current protocols for producing monocellular epithelial liver organoids, multi-tissue liver organoids, and multi-organ liver organoids in terms of their origins, cell types, self-renewal capacity, and functionality.

### 1) Liver organoids from primary tissues

Following the breakthrough report demonstrating the successful production of intestinal organoids 90, 91, studies have reported the production of liver epithelial organoids from primary human liver tissues 14, 92. Minced tissue fragments or even single cell-dissociated primary hepatocytes and cholangiocytes have successfully produced liver epithelial organoids representing the tissue-specific characteristics of either hepatocytes or cholangiocytes, resulting in the establishment of self-renewing hepatocyte organoids (HOs) and cholangiocyte organoids (COs), respectively 14, 93 (Figure 2).

COs can be further segregated into intrahepatic COs (ICOs) and extrahepatic COs (ECOs) based on their tissue of origin 94-96. Organoids typically closely resemble the molecular, functional, and morphological features of the tissues from which they are derived 97. However, COs display more dynamic cellular plasticity than organoids from other tissues 95. Indeed, mouse ICOs express not only cholangiocyte markers but also markers for progenitors and hepatocytes, suggesting their bipotential characteristics 92. Like mouse ICOs, human ICOs exhibit the concomitant expression of progenitor, hepatocyte, and cholangiocyte markers 14. Upon further differentiation, human ICOs acquire mature hepatocyte features, as evidenced by a series of *in vitro* functionality assays such as the secretion of albumin and bile acid, glycogen storage activity, and potential for detoxification and drug metabolism 14. In contrast to ICOs, ECOs could not activate hepatocyte-specific transcriptional program even with the same culture condition that can induce the transdifferentiation of ICOs into a hepatic state, showing the distinct cellular plasticity of COs based on their tissue of origin 94, 96, 98.

The establishment of stably expandable HOs from both primary mouse and human liver cells has also been demonstrated 99. HOs from both mouse and human exhibit morphology and gene expression patterns typical of hepatocytes but not cholangiocytes 93. Like ICOs, mouse HOs exhibit bipotential transdifferentiation capacity into either a hepatocyte or cholangiocyte state based upon the culture conditions 100. In contrast to human COs and mouse HOs, HOs from human liver cells show limited self-renewal capacity, necessitating the development of a new culture condition supporting the long-term expansion of human HOs 93. Nevertheless, HOs from both mouse and human are functionally mature, as shown by morphology typical of HOs, *in vitro* functionality, and the presence of bile canaliculi network 93.

### 2) Liver organoids from hPSCs

Due to the limited accessibility of primary tissue, pluripotent stem cells (PSCs) including embryonic stem cells (ESCs) and induced pluripotent stem cells (iPSCs) have been highlighted as alternative sources for organoid production 101, 102. Indeed, the production of organoids with structural and functional similarities to distinct human organs have been well documented over the past decade. Furthermore, in contrast to adult tissue-derived organoids, PSC-derived organoids hold great benefits for patient-specific disease modeling and drug discovery 101.

#### A. Monocellular liver organoids from PSCs

There are also several protocols available for differentiating PSCs into HOs 25, 28, 29, 103, 104. In contrast to HOs from primary liver tissue, PSC-derived HOs are morphologically similar to COs from primary tissues, as they grow in epithelial cyst form 25, 103-105. PSC-derived HOs are stably expandable *in vitro*, and display key hepatic functions upon maturation 25, 103-105. Furthermore, their usefulness as an *in vitro* disease modeling platform has been validated for steatosis and citrullinemia, a rare autosomal recessive genetic disorder 25, 105. Using a novel 3D protocol, Guan et al. showed the production of HOs with interestingly diverse morphologies 104. Their HOs are composed of either hepatocytes, cholangiocytes, or a mixture of both hepatocytes and cholangiocytes with self-renewal capacity. The HOs are functionally mature, as evidenced by glycogen storage, drug metabolism, and secretion of albumin and bile acids 103. Moreover, this previous work employed the researchers' HOs for modeling Alagille syndrome, a rare genetic disorder characterized by paucity of bile ducts, and patient-derived HOs were found to exhibit fewer duct-like structures as in the patients' liver 104.

The differentiation of PSCs including both ESCs and iPSCs into COs has also been described 106-108. PSC-derived COs are morphologically and functionally similar to COs from primary tissue and also form branched tubular structures 106. PSC-derived COs with key cholangiocyte functions could be used for modeling genetic diseases that cause hepatobiliary complications, such as cystic fibrosis and Alagille syndrome 109, 110. Furthermore, pathological phenotypes could be rescued by pharmacological intervention, suggesting that liver organoids are a promising tool for not only disease modeling but also drug screening.

Recent studies have successfully recapitulated the functional interconnection between hepatocytes and cholangiocytes 29, 111. These recent works utilized pre-differentiated hepatoblasts as a starting population to achieve the robust and homogeneous production of HOs. The generated HOs displayed a unique structural feature in which a dense hepatic core is surrounded by multiple biliary cysts. Both hepatocytes and cholangiocytes in HOs were found to be functional, as shown by the secretion of albumin and apolipoprotein B and activity of gamma glutamyl transferase and alkaline phosphatase, respectively. Furthermore, both the hepatic and bile duct parts are functionally interconnected through a bile canaliculi network, and this unique and advanced structure is suitable for modeling drug-induced cholestasis 29.

Collectively, different types of liver organoids (e.g., HOs, COs, and even HOs with functional interconnection between hepatic and biliary structures) could be generated from PSCs, and each type of liver organoid represents a suitable *in vitro* model system for studying distinct liver diseases.

#### B. Multi-tissue liver organoids from PSCs

The first step of liver organogenesis is the formation of the liver bud, a condensed structure in which primitive hepatic endoderm cells from the foregut endodermal sheet delaminate and invade the septum transversum mesenchyme, which is the source of HSCs as well as LSECs that begin to form vessels 112-114. These dynamic morphogenetic changes are orchestrated by nascent endothelial cells and adjunct cardiac mesoderm. Using classical-guided differentiation protocols, PSCs normally produce cell types from a singular germ layer, despite their pluripotency 115. In contrast, unguided differentiation protocols based on intrinsic differentiation signals allow for differentiation of PSCs into relatively diverse cell types from multiple germ layers 116. The differentiation procedure of liver organoids is highly defined and composed of multiple differentiation steps tightly guided by signal pathways 117. Both hepatocytes and cholangiocytes originate from the same source, hepatoblasts, that could be differentiated from definitive endoderm 118, 119. However, mesoderm is the common origin of non-parenchymal cells such as Kupffer cells, HSCs, and LSECs 120-122. Thus, achieving multi-tissue liver organoids containing both endoderm-derived parenchymal cells (e.g., hepatocytes and cholangiocytes) and mesoderm-derived non-parenchymal cells (e.g., Kupffer cells, HSCs, and LSECs) is theoretically and technically challenging.

By recapitulating organogenetic interactions between endothelial and mesenchymal cells *in vitro*, Takebe et al. 123 have successfully demonstrated the generation of multi-tissue liver organoids, so-called liver bud containing both endoderm- and mesoderm-derived cell types by coculturing iPSC-derived hepatic endodermal cells with human umbilical vein endothelial cells and mesenchymal stem cells (Figure 2). This *in vitro* reconstructed liver bud displays similarity with the *in vivo* liver bud in terms of gene expression patterns and vascularized structures. Upon transplantation into a cranial window model, the human vasculatures in the engrafted liver bud could functionally be connected to the host vessels, contributing to the functional maturation of the engrafted liver bud.

Instead of coculturing parenchymal cells and non-parenchymal supporting cell types, Ouchi et al. induced the concomitant differentiation of PSCs into multiple germ layers 28. Although the researchers' protocol is based on guided differentiation, they tried to implement a mesodermal differentiation cue by adding retinoic acid, which plays dual roles for both parenchymal and non-parenchymal cell specification (Figure 2). The resultant multi-tissue liver organoids contain not only parenchymal hepatocytes and cholangiocytes but also non-parenchymal supporting cells such as Kupffer cells and HSCs under the same culture conditions. Interestingly, both Kupffer cells and HSCs in these multi-tissue liver organoids are functional, as demonstrated by proper immune response against inflammatory stimuli and vitamin A storage activity, respectively. Furthermore, both the activation of HSCs and excessive production of ECM could be observed under steatosis conditions. Taken together, these results show that multi-tissue liver organoids containing non-parenchymal supporting cell types may be an advanced platform for closely mirroring the *in vivo* scenario, allowing for more precise disease modeling and drug screening for liver diseases.

#### C. Multi-organ liver organoids from PSCs

Close interactions among distinct organs are essential for maintaining the diverse vital functions of the human body. For example, the liver is tightly linked to the pancreas, and the aberrant interactions between the two organs may lead to dysregulated glucose levels and metabolic disorders such as type 2 diabetes mellitus 124. Thus, recapitulating inter-organ interactions *in vitro* is important for closely simulating the real *in vivo* situation as well as discovering potential therapeutics for diseases such as type 2 diabetes mellitus mediated by altered inter-organ interactions. To this end, several previous studies have attempted to reproduce the *in vivo* interactions between distinct tissue compartments using organoid technology. Indeed, recent advances in organoid technology have successfully demonstrated the generation of assembloids between distinct brain organoids representing different parts of brain tissues 125. Moreover, the potential usefulness of assembloid technology has been well described in previous studies 126, 127 wherein pathological outputs that could be mediated by the interactions among distinct parts of brain were successfully recapitulated in patient-derived assembloids.

Recently, the generation of multi-organ liver organoids with functional interconnection to biliary and pancreatic domains was also demonstrated by fusing anterior gut spheroids with posterior gut spheroids derived from human PSCs 89. Previous research conducted by Koike et al. involved the fusion of anterior and posterior spheroids to generate boundary organoids containing multi-endoderm domains. Through cell-to-cell communication, hepato-biliary-pancreatic (HBP) progenitors expressing HHEX and PDX1 emerged at the interface of the fused spheroids. The specification of HBP progenitors in the fused spheroids was found to be critically influenced by retinoic acid signals. Isolated HBP progenitor domains from the fused spheroids, when cultured for an extended period, developed into mature organoids known as HBP organoids. The long-term culture of HBP organoids demonstrated the presence of multiple organ domains comprising hepato-biliary-pancreatic domains, with an interconnected functioning between the pancreas and bile duct. It is worth noting that the abolishment of HES1, a transcription factor regulating the posterior foregut lineage 128, 129, could lead to the conversion of biliary tissue into pancreatic tissue in mice 130, 131, with an increased number of pancreatic structures observed in HES1-deficient HBP organoids, suggesting the potential utility of multi-organ liver organoids for closely recapitulating human organogenesis and facilitating *in vitro* disease modeling 89.

Combined with state-of-art tissue engineering technologies, such as organ-on-chip technology, inter-organ interactions could also be recapitulated in liver organoids. Indeed, a recent study has successfully demonstrated the recapitulation of human liver-pancreatic islet axis using a microfluidic multi-organoid system 132. Dynamic interaction between two organoids, as evidenced by glucose-stimulated insulin secretion from islet organoids and altered glucose utilization in liver organoids, suggests the potential usefulness of multi-organ liver organoids for both studying the complex pathogenesis of metabolic disorders and developing new therapeutics 132. Further efforts for recapitulating more complex and mature inter-organ crosstalk in liver organoids are required for an advanced platform for *in vitro* disease modeling and drug discovery.

## 3. Customized liver organoids for distinct therapeutic targets

For the study of NAFLD and NASH, animal models, particularly murine models, have been extensively employed to elucidate the underlying mechanisms of pathogenesis. However, the use of larger animal models such as rabbits, minipigs, and monkeys, which are more similar to humans, has been limited due to ethical concerns, difficulties in handling, time requirements, and high costs 133, 134. Three primary types of murine models, namely genetic, dietary, and combination models, have been widely used for replicating the pathophysiology of human NAFLD and NASH (Table 1) 135. An ideal animal model for NAFLD/NASH should exhibit not only steatosis (accumulation of fat in the liver) but also steatohepatitis phenotypes, including inflammation and fibrosis 136. Furthermore, considering the close association between metabolic disorders and NAFLD/NASH, an ideal animal model should replicate the metabolic abnormalities observed in NAFLD/NASH patients 137-140. Genetic models, such as SREBP-1c transgenic mice and PTEN null mice, exhibit phenotypes charactertistic of both steatosis and steatohepatitis 141-143. On the other hand, genetic models like ob/ob mice, db/db mice, and KK-Ay mice primarily display steatosis phenotypes, without progressing to steatohepatitis in the absence of additional factors like a high-fat diet 144-148. This discrepancy in pathological patterns among different animal models suggests that the diverse outcomes may hinder the translation of findings from animal models to clinical applications. Dietary animal models, on the other hand, succeed in relatively replicating steatohepatitis phenotypes 149, although the specific pathological outcomes vary depending on species, strain, and gender 150. To overcome these challenges, combination models, which involve feeding specific diets to genetic murine models, have been employed and have demonstrated close resemblance to human diseases 135, 151, 152. Nevertheless, it is important to acknowledge the significant species-specific differences between human and animal livers, which may impede the clinical translation of discoveries made in animal models. Furthermore, animal models often fail to fully recapitulate the entire spectrum of NAFLD and NASH observed in humans 135, 153, 154.

For *in vitro* modeling of NAFLD and NASH, the 2D monolayer cell culture system using singular hepatic cell types has been widely adopted due to its relatively low cost, easy handling, and high- compatibility with high-throughput applications (Table 1). Due to the limited availability and rapid loss of functionality of PHHs, alternative cell sources, including immortalized cell lines (HepG2, HuH-7, and HepaRG) and hPSC-derived 2D hepatocyte-like cells, have been proposed as substitutes for PHHs. Previous studies 155-162 have successfully demonstrated that the 2D monolayer cell culture system could successfully recapitulate hallmarks of NAFLD and NASH, including cytoplasmic accumulation of triglycerides in hepatocytes, ER stress, inflammation, and cell death. Consequently, the 2D monolayer cell culture system has been used for evaluating drug-induced hepatotoxicity and the efficacy of therapeutic compounds. However, the 2D monolayer cell culture models have limitations, as they lack non-parenchymal cell types, which play a key role in the pathogenesis of NAFLD and NASH 163. Additionally, altered metabolic functionality has been another challenging issue of 2D monolayer cell culture models 164. Furthermore, the inability to replicate cell-to-cell and cell-to-matrix interactions as well as biomolecular gradients present in tissues, hinders the accurate representation of physiological conditions in the 2D monolayer cell culture system 24. Therefore, more sophisticated culture systems that mimic *in vivo*-like cellular compartments and spatial organization are required. Coculturing hepatocytes with the missing non-parenchymal cell types has shown significant improvements in the functional aspects of *in vitro* NAFLD and NASH models 165-167. However, further efforts are needed for obtaining a sufficient number of highly pure and functional non-parenchymal cell populations, as well as optimizing the coculture condition for maintaining distinct cell types using a singular medium condition, before coculture models can be fully established.

As we discussed in the first section, the cellular crosstalk among distinct cellular compartments including both parenchymal and non-parenchymal cell types is critical for the pathogenesis of NAFLD and NASH. Indeed, each cell type plays crucial and diverse roles for the induction and progression of liver diseases. For this reason, PHHs and 2D hepatocyte-like cells are insufficient for reproducing the cellular crosstalk, as they lack pro-inflammatory and pro-fibrotic cell types, which are critical for initiation and progression of NAFLD and NASH 24, 168. Animal models also show fundamental genetic and physiological differences with humans 169. Therefore, recent liver organoids that exhibit structural and functional similarity to liver tissue may represent an advanced and attractive human-specific model system 27, 28. However, as we described in the second section, substantial differences in current liver organoid technologies contribute to the variability of generated liver organoids in terms of origin, cellular compartment, functionality, self-renewal capacity, and most importantly capacity for reproducing inter-cellular, inter-tissue, and inter-organ communications. Although this variability may be a hurdle to overcome for achieving the standardized production of highly uniform organoids, it also provides a great opportunity for utilizing customized liver organoids with distinct inter-cellular, inter-tissue, and inter-organ communications as a novel *in vitro* model system for precisely predicting the efficacy of diverse drug candidates targeting distinct therapeutic targets. In this last section, we discuss how to utilize distinct types of liver organoids including monocellular epithelial liver organoids, multi-tissue liver organoids, and multi-organ liver organoids for unveiling the mechanism underlying the pathogenesis of NAFLD and NASH as well as for evaluating the efficacy of drug candidates targeting distinct therapeutic mechanisms.

### 1) Driving force to NAFLD/NASH and related therapeutic targets

Currently, lifestyle modification has been the first-line treatment for preventing and controlling NAFLD and NASH 170. However, lifestyle intervention is not a viable treatment option in patients with advanced fibrosis or cirrhosis 171. Thus, the development of novel treatments that could effectively and safely reverse NASH symptoms including fibrosis are urgently demanded. Although lipogenesis, by the increased delivery of FFAs from diet and adipose tissue into the liver or the increased *de novo* lipogenesis, has been considered as a major factor for the pathogenesis of NAFLD, growing evidence suggests that NASH is rather mediated by the synergistic interaction among multiple concomitant factors such as genetic variants, metabolic disorders, oxidative stress, altered immune response, and even the disruption of the gut-liver axis 41. Considering this complex mechanism underlying the development of NASH, diverse treatment strategies for NASH with distinct therapeutic targets are currently under preclinical and clinical trial testing.

### 2) Customized organoids for each therapeutic target

As the outcomes of current liver organoid technology look quite diverse in terms of cellular compartments, appropriately generated liver organoids containing the right cell types with proper inter-cellular, inter-tissue, and inter-organ crosstalk are prerequisites for evaluating the efficacy of each drug candidate targeting distinct pathological mechanisms. Here we suggest that different types of liver organoids are theoretically suitable for evaluating current drug candidates in different stages of clinical trials based on their target mechanism such as *de novo* lipogenesis, metabolism, cellular stress, inflammation, and fibrosis. Based on this, we suggest a concept of utilizing customized liver organoids with distinct inter-cellular, inter-tissue, or inter-organ interactions, which might have a great potential for evaluating drug candidates targeting distinct mechanisms underlying the development and progression of NAFLD and NASH.

#### A. Therapeutic targets requiring monocellular epithelial liver organoids

**Figure 3:**
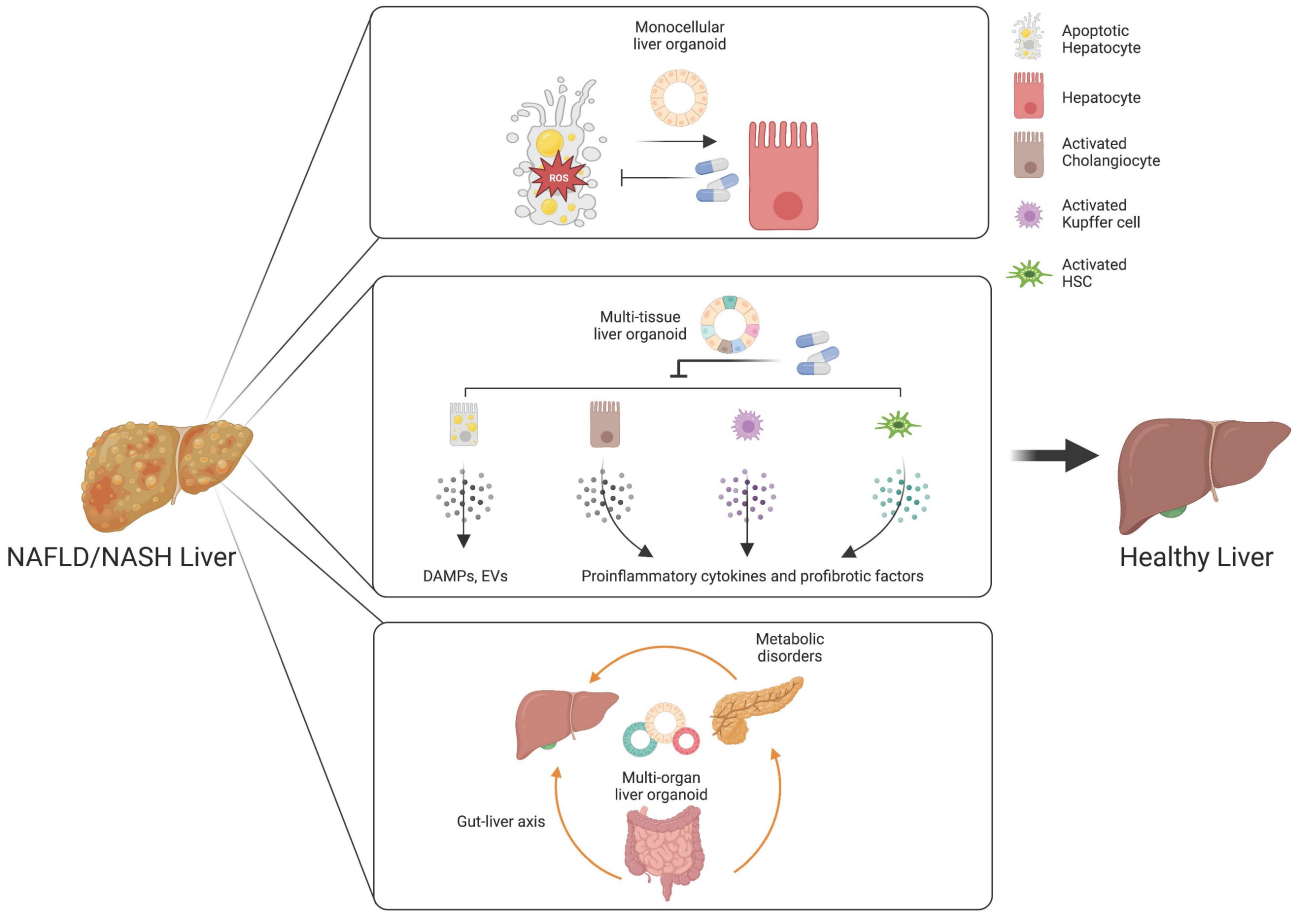
Customized liver organoids for evaluating the efficacy of drugs targeting distinct therapeutic targets. Monocellular liver organoids can be a useful model for drug candidates targeting *de novo* lipogenesis, anti-cellular stress, and hepatic cell death. Multi-tissue liver organoids are suitable for drugs targeting an inflammatory response and hepatocyte-derived DAMPS or EVs. Multi-organ liver organoids may serve as a great model for understanding the influences of the gut-liver axis and metabolic disorders in the pathogenesis of NAFLD and NASH.

Monocellular epithelial liver organoids, such as HOs and COs both from tissue biopsy and PSCs, consist mostly of a single cell type 14, 25, 26. Although epithelial HOs and COs recapitulate the structural features of liver tissue in a relatively limited way compared with multi-tissue liver organoids, they can self-renew and become fully functional upon further maturation 25. Despite the limited cellular diversity of monocellular epithelial liver organoids, these organoids can be a useful model for efficacy evaluation for some drug candidates targeting *de novo* lipogenesis, anti-cellular stress, and hepatic cell death (Figure 3) 172.

*De novo* lipogenesis is the primary factor associated with fatty liver 173. Indeed, increased *de novo* lipogenesis is observed in 20% to ~30% of patients with NAFLD and NASH compared to unaffected individuals 174. Both key transcription factors such as SREBP-1c (sterol regulatory element-binding protein 1c) and ChREBP (carbohydrate regulatory element-binding protein) and enzymes including acetyl-CoA carboxylase (ACC) and fatty acid synthase are involved in *de novo* lipogenesis and serve as potential therapeutic targets 173. Currently, diverse drug candidates are in both preclinical and clinical trial testing; these candidates inhibit: 1) ATP-citrate lyase (ACLY), a cytoplasmic enzyme responsible for the generation of acetyl-coenzyme A (acetyl-CoA); 2) ACC, which converts acetyl-CoA to malonyl-CoA; 3) FAS, a rate-controlling enzyme that converts malonyl-CoA into palmitic acid during *de novo* lipogenesis; 4) SCD1 (stearoyl coenzyme A desaturase 1), an enzyme that catalyzes the rate-limiting step in the formation of monosaturated fatty acids; 5) SREBP-1c, an insulin-sensitive transcription factor that plays a key role in the induction of lipogenic genes in the liver; and 6) SREBP2, a key transcription factor regulating expression of genes involved in cholesterol biosynthesis 175. Although all the inhibitors aim at distinct targets during lipogenesis, they all require a liver organoid model by which the distinct steps of lipogenesis can be effectively inhibited for reducing hepatic *de novo* lipogenesis and steatosis. Thus, monocellular epithelial liver organoids such as HOs and COs may be a suitable liver organoid model for drug candidates targeting lipogenesis. Indeed, monocellular epithelial liver organoids from human fetal liver have recently been used for *in vitro* modeling of steatosis and for finding potential drug candidates for NAFLD. In a study by Hendriks et al. 176, 17 candidate NAFLD drugs from recent drug development programs were screened using monocellular epithelial liver organoids. Among them, inhibitors of ACC, FAS, DGAT2 (diacylglycerol O-acyltransferase 2), FXR (Farnesoid X receptor) agonists, and recombinant FGF19 were found to effectively reduce the steatosis phenotype, thus supporting the concept that monocellular epithelial liver organoids serve as a suitable model for drug candidates targeting *de novo* lipogenesis.

Oxidative stress, a condition by which the generation of highly toxic ROS exceeds the capacity of antioxidants to detoxify, is also a major factor in the pathogenesis of several chronic diseases including NAFLD 177. The accumulation of lipids leads to overproduction of ROS and stress in mitochondria and ER, contributing to inflammation, cellular injury, and cell death 178. Several enzymes involved in the detoxification process of ROS and diverse antioxidants including vitamin C (ascorbic acid), vitamin A (retinol), and vitamin E (tocopherol) may be a therapeutic target for reducing ROS 179. Currently, several antioxidants including vitamin E are under investigation to address their therapeutic effects for NAFLD and NASH 180. Therefore, monocellular epithelial liver organoids including HOs and COs may also be a desirable model for evaluating the efficacy of antioxidants as drug candidates for NAFLD and NASH.

NASH is also characterized by hepatocyte injury, and thus cell death seems to be an important factor in the progression of NAFLD and NASH 181. It has also been suggested that distinct cell death mechanisms including apoptosis, necroptosis, pyroptosis, ferroptosis, and autophagy are associated with progression of NAFLD and NASH 182. Among the distinct cell death pathways, apoptosis is the most common and best-characterized cell death pathway in NASH 183. Indeed, diverse anti-apoptotic agents including Emricasan, Selonsertib, and Rapamycin attenuate inflammation and reverse fibrosis during preclinical studies 184-186. Besides apoptosis, a growing body of evidence suggests that necroptosis, pyroptosis, and ferroptosis may play a crucial role in the development of NAFLD and NASH, thus expanding the potential therapeutic targets to diverse cell death pathways 182. Again, monocellular epithelial liver organoids may be an appropriate model for evaluating anti-cell death agents.

#### B. Therapeutic targets requiring multi-tissue liver organoids

An inflammatory response is a prerequisite for the initiation and progression of NAFLD and NASH 187. Immune cells and pro-inflammatory cytokines play crucial roles in the pathogenesis of NAFLD and NASH 188. As we already described in the first section, Kupffer cells are the key cell type for regulating and maintaining immunity in the liver. Upon liver injury, the damaged hepatocytes lead to activation of Kupffer cells and infiltration of circulating monocyte-derived macrophages 189. The activated macrophages produce a plethora of pro-inflammatory cytokines, resulting in the activation of HSCs 61. Activated HSCs are also known to produce pro-inflammatory chemokines that attract monocytes into the injured liver 190. In pathological conditions, cholangiocytes also play an important role in liver inflammation via secretion of cytokines and fibrotic factors 191. Hepatocyte-derived DAMPs are also known to be a major mediator for immune response 192. Moreover, the tight crosstalk among distinct cellular compartments is critical for the ignition and further progression of NAFLD and NASH, as previously discussed. Taken together, these findings show that the inflammatory response produced during the initiation and progression of NAFLD and NASH is orchestrated by multiple cellular compartments and their tight crosstalk in the liver. In this case, multi-tissue liver organoids containing major pro-inflammatory cell types including DAMP-producing hepatocytes, activated cholangiocytes, activated Kupffer cells, and activated HSCs would be beneficial for recapitulating the *in vivo* pro-inflammatory immune response and for evaluating the therapeutic efficacy of drug candidates (Figure 3).

The damaged hepatocytes and activated Kupffer cells lead to the activation and transdifferentiation of HSCs into ECM-producing myofibroblasts 193. Thus, multiple drug candidates either directly affecting ECM synthesis and turnover or indirectly influencing HSC activation via inhibition of TGF-β, the most potent fibrotic factor, are currently under preclinical and clinical investigation 194. In this case, multi-tissue liver organoids containing Kupffer cells and HSCs might also be a useful model.

Extracellular vesicles (EVs) have been highlighted as a novel therapeutic target for NAFLD and NASH 195. EVs play an important role in intercellular communication as a signaling mediator between liver and other organs by carrying various bioactive molecules including lipids, proteins, DNA, and RNAs 195. The increased level of hepatocyte-derived EVs correlates with the severity of NASH, and thus the protein and mRNA composition of these EVs may be a useful biomarker for the diagnosis of NAFLD and NASH 196. In NAFLD and NASH, EVs lead to lipid accumulation and activation of macrophages and HSCs, promoting inflammation and fibrosis 197. Therefore, EVs may serve as new targets for the treatment of NAFLD and NASH. In this sense, multi-tissue liver organoids containing both donors and recipients of EVs would be a suitable model for unveiling the role of EVs in the pathogenesis of NAFLD and NASH.

Recently, Ouchi et al. 198 established a multi-tissue liver organoid-based steatosis model using either FFA exposure or Wolman disease patient-derived iPSCs and successfully replicated typical symptoms of NAFLD and NASH, including lipid accumulation, triglyceride production, hepatocyte ballooning, increased stiffness, proliferation of HSCs, production of inflammatory cytokine, recruitment of immune cells, and enhanced fibrosis. Treatment with recombinant FGF19 was found to signicantly reduce the pathological hallmarks of NASH in the multi-tissue liver organoid-based steatosis model, such as lipid accumulation, hepatocyte damage, stiffness, and ROS production. Another study by Guan et al. 199 used similar multi-tissue liver organoids to model autosomal recessive polycystic kidney disease (ARPKD), a monogenic disorder that causes liver fibrosis. Severe fibrosis was observed in ARPKD liver organoids, mediated by the activation of HSCs, and was efficiently ameliorated by treatment with PDGFR tyrosine kinase inhibitors (Crenolanib, Sunitinib, Imatinib). These previous reports strongly support the notion that multi-tissue liver organoids provide a suitable model for replicating typical symptoms of NAFLD and NASH, as well as for evaluating the efficacy of drug candidates targeting therapeutic mechanisms mediated by inter-tissue interactions.

#### C. Therapeutic targets requiring multi-organ liver organoids

The gut-liver axis supports bidirectional interactions between the liver and gastrointestinal tract, where trillions of microorganisms form the gut microbiota 200. The bile acids synthesized from the liver influence the composition and function of the gut microbiota in the gastrointestinal tract 201. On the other side, the gut microbiota and its metabolites regulate the synthesis of bile acids and hepatic lipid metabolism 202. The gastrointestinal tract, the largest mammalian-microbial interface, regulates symbiotic interactions between the host and microorganisms 203. In a physiologically healthy condition, the gastrointestinal epithelial barrier supports digestion, immunity, and metabolic function 204. Another important role of the intestinal barrier entails preventing the entrance of harmful intestinal bacteria and their metabolites into the circulation 205. However, increased epithelial permeability mediated by disruption of the gastrointestinal epithelial barrier leads to bacterial translocation, which promotes hepatic inflammation and oxidative stress, contributing to the pathogenesis of NAFLD and NASH 206. Multi-organ liver organoids may serve as a great *in vitro* model for reproducing the gut-liver axis and for developing a novel class of drugs targeting a disturbed gut-liver axis (Figure 3).

NAFLD and NASH are closely associated with metabolic disorders including type 2 diabetes mellitus 207. The global prevalence of NAFLD and NASH in patients with type 2 diabetes mellitus surpasses 55% 208. Currently, diverse antidiabetic drugs for NAFLD and NASH are in clinical trial testing and are exhibiting promising efficacy 209. Similarly, multi-organ organoids that reproduce the pathology of both metabolic disorders and chronic liver diseases may be a useful model for evaluating the efficacy of antidiabetic drug candidates.

## 4. Conclusion

The scientific society has reached a consensus that liver organoid technology is an advanced and suitable human-specific model system for closely recapitulating the liver *in vitro*. Nevertheless, many hurdles remain to be addressed before translating liver organoid technology into the clinic and industrial settings. First, many protocols have failed to fully reproduce all the cellular compartments in the liver. Although recent advances have described the generation of multi-tissue liver organoids containing relatively diverse cell types 27, 28, the presence of functional LSECs with their proper structural and functional features has yet to be reported. Moreover, creating liver organoids containing all the parenchymal and non-parenchymal cell types in a ratio similar to liver tissue is paramount for recapitulating the cellular crosstalk that occurs* in vivo*. Second, there is no standardized protocol for liver organoid production, perhaps appropriating much time and valuable resources of many laboratories for simply reproducing some leading protocols. Indeed, the resultant liver organoids generated by different labs display quite distinct features, even with similar differentiation guiding cues, bringing the issue of reproducibility to the forefront. Third, as with other types of organoids, the variation between batches of liver organoids or even between individual liver organoids is a critical consideration impeding the translation of liver organoid technology. Fourth, the unique structure of the liver has not been demonstrated *in vitro*. Although multiple studies have successfully described the structural similarity of liver organoids with liver tissue by demonstrating the presence of functional bile canaliculi networks within liver organoids 25, 29, 103, 104, the production of liver organoids showing the typical hexagonal hepatic lobules consisting of a portal triad remains challenging. Nevertheless, liver organoids represent an alternative technology with the potential for addressing and overcoming the diverse limitations of preexisting animal models and 2D cell culture systems toward an enhanced understanding of the mechanisms underlying liver diseases as well as drug discovery and efficacy and safety testing 24.

Of course, a future direction of liver organoid technology should aim to fully reproduce the inter-cellular, inter-tissue, and even inter-organ communications that occur in the human body. Microfluidic-based organ-on-chip technology, together with standardized liver organoid production technology, may facilitate this concept 210, 211. However, current liver organoid technology is able to recapitulate only relatively limited crosstalk across distinct cell types, tissues, and organs. We therefore suggest to utilize distinct types of liver organoids such as monocellular epithelial organoids, multi-tissue organoids, and multi-organ organoids that reflect the distinct causes of NAFLD and NASH to facilitate our fundamental understanding of underlying disease mechanisms as well as accelerate the development of effective treatments for NAFLD and NASH.

## Figures and Tables

**Table 1:** Comparison of animal models, 2D monolayer cell culture models, and 3D liver organoid models.

Model	Species	Type	Phenotype reproduced	Advantages	Disadvantages
Animal models	Animal	SREBP-1c transgenic mice (141, 212)	Steatosis, Insulin resistance, Inflammation, Fibrosis	-Physiological environment -Immune system -Multiple cell types -Functionality -Structure -Metabolism	-Non-human species -Low-throughput -High cost -Heterogeneous phenotypes (species, strain, gender)
		PTEN null mice (142, 143)	Steatosis, Fibrosis		
		Ob/ob mice (144, 213, 214) Db/db mice (146, 147, 215)	Obesity, Steatosis, Insulin resistance, (Inflammation, Fibrosis)		
		MAT1A null mice (216-218)	Steatosis, Fibrosis		
		High-fat diet mice (150, 219, 220)	Obesity, Steatosis, Hepatic insulin resistance, Oxidative stress, Inflammation, Fibrosis		
		Methionine- and choline-deficient diet mice (221-223)	Steatosis, Hepatic Insulin resistance, Inflammation, Oxidative stress, Mitochondrial damage, Apoptosis, Fibrosis		
		Cholesterol and cholate diet mice (224)	Steatosis, Hepatic Insulin resistance, Inflammation, Oxidative stress, Fibrosis		
2D monolayer cell culture models	Human	PHHs (155, 225)	Steatosis, ER stress, Apoptosis	-Low cost -Easy handling -High-throughput compatibility -Easy downstream application	-Limited physiological environment -Absence of non-parenchymal cell types - Absence of cell-to-cell and cell-to-matrix interactions -Low hepatic maturity
		HepG2 (159, 226, 227)	Steatosis, Apoptosis,		
		HuH 7 (159, 160, 228)	Steatosis, ER stress, Apoptosis,		
		HepaRG (161, 229, 230)	Steatosis, Oxidative stress		
		PSC-hepatocyte-like cells (162, 231)	Steatosis		
		PSC-HSCs (232)	Inflammation, Activation of HSCs		
3D liver organoid models	Human	Spheroids (3D coculture) (233-235)	Steatosis, Activation of HSCs, Oxidative stress, Apoptosis, Inflammation, Expression of profibrotic markers, Mitochondrial dysfunction	-Complex structural organization -Long-term expansion -Multiple cell types -High/mid-throughput compatibility -Semi-physiological environment -Immune system	-Complicated differentiation steps -Relatively high cost -Heterogeneity (batch variation) -Limited hepatic maturity
		Monocellular liver organoids (from primary tissue) (176, 236, 237)	Mainly steatosis		
		Multi-tissue liver organoids (198, 199, 238)	Steatosis, HSC activation, Ductular reaction, Oxidative stress, Bile canaliculi disruption, Expression of profibrotic markers, Collagen secretion and deposition		
